# Encoding type, medication, and deep brain stimulation differentially affect memory-guided sequential reaching movements in Parkinson's disease

**DOI:** 10.3389/fneur.2022.980935

**Published:** 2022-10-17

**Authors:** Fabian J. David, Yessenia M. Rivera, Tara K. Entezar, Rishabh Arora, Quentin H. Drane, Miranda J. Munoz, Joshua M. Rosenow, Sepehr B. Sani, Gian D. Pal, Leonard Verhagen-Metman, Daniel M. Corcos

**Affiliations:** ^1^Department of Physical Therapy and Human Movement Sciences, Northwestern University Feinberg School of Medicine, Chicago, IL, United States; ^2^School of Integrative Biology, University of Illinois at Urbana-Champaign, Urbana-Champaign, IL, United States; ^3^Department of Neurological Surgery, Northwestern University Feinberg School of Medicine, Chicago, IL, United States; ^4^Department of Neurosurgery, Rush University Medical Center, Chicago, IL, United States; ^5^Department of Neurology, Rutgers University, New Brunswick, NJ, United States; ^6^Department of Neurology, Northwestern University Feinberg School of Medicine, Chicago, IL, United States

**Keywords:** encoding, Parkinson's disease, proprioception, peripheral-vision levodopa, subthalamic nucleus deep brain stimulation (STN-DBS)

## Abstract

Memory-guided movements, vital to daily activities, are especially impaired in Parkinson's disease (PD). However, studies examining the effects of how information is encoded in memory and the effects of common treatments of PD, such as medication and subthalamic nucleus deep brain stimulation (STN-DBS), on memory-guided movements are uncommon and their findings are equivocal. We designed two memory-guided sequential reaching tasks, peripheral-vision or proprioception encoded, to investigate the effects of encoding type (peripheral-vision vs. proprioception), medication (on- vs. off-), STN-DBS (on- vs. off-, while off-medication), and compared STN-DBS vs. medication on reaching amplitude, error, and velocity. We collected data from 16 (analyzed *n* = 7) participants with PD, pre- and post-STN-DBS surgery, and 17 (analyzed *n* = 14) healthy controls. We had four important findings. First, encoding type differentially affected reaching performance: peripheral-vision reaches were faster and more accurate. Also, encoding type differentially affected reaching deficits in PD compared to healthy controls: peripheral-vision reaches manifested larger deficits in amplitude. Second, the effect of medication depended on encoding type: medication had no effect on amplitude, but reduced error for both encoding types, and increased velocity only during peripheral-vision encoding. Third, the effect of STN-DBS depended on encoding type: STN-DBS increased amplitude for both encoding types, increased error during proprioception encoding, and increased velocity for both encoding types. Fourth, STN-DBS was superior to medication with respect to increasing amplitude and velocity, whereas medication was superior to STN-DBS with respect to reducing error. We discuss our findings in the context of the previous literature and consider mechanisms for the differential effects of medication and STN-DBS.

## Introduction

Memory-guided reaching is vital to many daily activities: when reaching for the light switch when we wake up in the middle of the night, when reaching for the cup holder while driving, or when reaching for a computer mouse. It belongs to a class of goal-oriented movements referred to as internally generated movements because their execution does not rely on external feedback; instead, they rely on information stored in memory (1–5). Thus, fundamental to memory-guided reaching is the reliance on sensory inputs to encode information in memory to enable the planning and execution of movement. Often, visual (including peripheral-vision) and/or proprioceptive sensory inputs are used both for encoding target locations into memory and for execution of memory-guided movements (6, 7). In persons with Parkinson's disease (PD), motor deficits are known to be accompanied by visual deficits, including peripheral-visual deficits (8, 9), and somatosensory deficits (10, 11). Peripheral-visual deficits reported in PD include greater distraction from peripheral objects (12), reduced responsiveness to peripheral events (9), and reduced contrast sensitivity (13). Somatosensory deficits include deficits in sensory perception and integration (11, 14, 15). Yet, the extent to which peripheral-visual or proprioceptive sensory inputs, i.e., different encoding types, affect reaching performance outcomes such as amplitude, error, and velocity remain to be compared in people with PD. This is important because these deficits can have a significant impact on memory-guided motor performance. If the sensory input and/or how it is integrated with the motor output is abnormal, then these sensory deficits might play a key role in any observed motor deficits. In addition, how one encodes information into memory can differentially affect motor performance. Therefore, a critical first step is to determine if there are differences in memory-guided reaching performance as a function of encoding type in people with PD.

Visual encoding utilizes the ventral stream, the “what” pathway, that courses through the occipitotemporal cortex to identify the stimulus, and the dorsal stream, the “where” pathway, namely the occipito-parietal-prefrontal branch of the dorsal stream, for spatial location and spatial working memory (16). On the other hand, proprioceptive encoding utilizes the sensorimotor cortex, the lateral premotor cortex, and the anterior cerebellum (17). In addition, there is evidence for visual occipital areas being engaged during proprioceptive reaches, likely due to “visualizing” the proprioceptively encoded targets (18). Furthermore, the basal ganglia-thalamo-cortical network is also involved in the planning and execution of memory-guided movements (19, 20). Cortical inputs to the striatum are topographically organized and functionally segregated (21–24). For instance, fronto-parietal association cortices that overlap with the occipito-parietal-prefrontal branch involved in spatial location and spatial working memory project to the caudate, while sensorimotor cortices project to the posterior putamen (21, 23). In addition, there is evidence that the tail of the caudate and adjoining ventral putamen receive projections from the inferior temporal visual cortex (25). Thus, cortical areas that process visuospatial information, including spatial working memory, and those that process somatosensory proprioceptive information project to separate areas in the striatum. As such, deficits in memory-guided reaching would be expected in persons with PD because striatal dopaminergic neuronal denervation alters the normal functioning of the basal ganglia-thalamo-cortical network. In fact, memory-guided and internally generated movements are especially impaired in patients with PD (26–28). Participants with PD, relative to healthy controls (HC), exhibit hypokinetic and bradykinetic movements with reduced amplitude (28) and/or reduced velocity (27), and with increased (29) or no difference in end-point error (28, 30). Reduced amplitude in PD, especially during memory-guided movements, has been associated with reduced striatal dopamine transporter binding, suggesting dependence on dopaminergic circuits (31). In PD, degeneration across the striatum is non-uniform (32, 33). Typically, the putamen degenerates earlier relative to the caudate (32, 33). Autopsy results show that dopaminergic loss is observed in all subdivisions of the putamen but only in the most dorsal rostral part of the caudate (33). The putamen is an integral part of the motor circuit, while the caudate is an integral part of the associative and limbic circuits (34). Consequently, motor symptoms and visuospatial deficits are likely to be the first symptoms to emerge, followed by cognitive and limbic symptoms (35). In addition, the pattern of degeneration might affect the response to treatments such as medication or sub-thalamic nucleus deep brain stimulation (STN-DBS). In theory, those areas that have already degenerated have greater dopaminergic deficit and are likely to respond beneficially to treatment, while those areas that have not yet degenerated are less likely to respond beneficially to treatment. This combination of topographic organization, functional segregation, and non-uniform progression of degeneration may not only lead to differential deficits in peripheral-visual and proprioceptively encoded reaching, but it may also differentially affect the response to dopamine replacement therapy and STN-DBS.

Studies examining the effect of treatments such as medication or STN-DBS on memory-guided reaching outcomes are surprisingly rare (36) despite the importance of memory-guided reaching in day-to-day activities, the key role that the basal ganglia play in the processing of memory-guided movements, and the fact that these movements are especially impaired in PD. We have shown that STN-DBS improves reaching outcomes of velocity while worsening error (36). Velocity is an intensive aspect of movement control requiring a simple scaling of gain, while error is a coordinative aspect and a more complex feature of movement control (37). STN-DBS facilitates intensive aspects while impairing coordinative aspects of control (36). The bulk of the literature examining the effect of medication or STN-DBS on internally generated movements that rely on spatial working memory is limited to eye movements (38–43). The findings with respect to the effect of medication are inconsistent. They show that medication either has no effect on amplitude (38–40) or worsens amplitude (41) and has no effect on velocity (38, 40). With respect to the effect of STN-DBS, the findings are more consistent. Most studies have shown a STN-DBS-induced increase in amplitude/gain (42–46), and one has shown an increase in velocity (38). Critically, to date, no study has systematically evaluated and compared the effect of treatment, i.e., medication and STN-DBS, on peripheral-visual vs. proprioceptively encoded memory-guided reaches on the same cohort of participants with PD, both prior to and after STN-DBS surgery. This kind of study on the same cohort will allow us to address questions related to the effect of medication, the effect of stimulation, and to compare the effect of medication and STN-DBS on peripheral-visual vs. proprioceptively encoded memory-guided reaching.

The purpose of this study was to address the gaps in our understanding with respect to the effects of encoding, anti-Parkinsonian medication, and STN-DBS on memory-guided sequential reaching in participants with PD. Toward this end, we designed two experimental tasks, one that utilized peripheral-visual encoding and another that utilized proprioceptive encoding to reach to remembered sequential spatial targets. We addressed the following gaps in our understanding: the effect of (1) encoding (peripheral-vision vs. proprioception), (2) medication (on vs. off), (3) STN-DBS (on vs. off), and (4) the differential effect of medication vs. STN-DBS on reaching amplitude, error, and velocity. We also compared the effect of medication vs. STN-DBS on the Movement Disorders Society Unified Parkinson's Disease Rating Scale motor score (MDS-UPDRS III), the gold standard to quantify motor signs of PD. We did this to determine if the findings related to our encoding types were consistent with findings from a well-established and standardized rating scale of motor signs of PD. Additionally, we provide data from age-matched healthy controls for comparison.

## Methods

### Participants

This study was conducted with approval from the Northwestern University and Rush University Medical Center Institutional Review Boards and with informed consent from all participants. Data were collected from 33 participants (PD: *n* = 16; HC: *n* = 17). Participants with PD were recruited from the Departments of Neurology/Neurological Sciences at Northwestern University (*n* = 1) and Rush University Medical Center (*n* = 15). A movement disorders neurologist examined all participants with PD, and they were included in the study if they: met the UK PD Society Brain Bank clinical diagnostic criteria for PD (47), were right-hand dominant, were able to understand and perform the experimental tasks, had normal or corrected visual acuity, presented no eye movement abnormalities such as double vision and/or blepharospasm, had no other neurological comorbidities, and had no orthopedic issues that could preclude completing experimental tasks. HC had no reported history of neurological disorders and had a score of ≤6 on the Movement Disorders Society Unified Parkinson's Disease Rating Scale Part III motor score (MDS-UPDRS III) (48). The cut-off of ≤6 for HC was determined from the mean MDS-UPDRS III (+2 standard deviations) from healthy controls (*n* = 196) who were part of the Parkinson's Progression Markers Initiative database (48). Apart from these two criteria, HC met the same inclusion/exclusion criteria as those with PD. The Edinburgh Handedness Inventory (49) was used to confirm right hand dominance, as participants used their right hand to complete the experimental tasks.

### Experimental conditions

Pre-STN-DBS, data collection for PD participants took place over 3 days. Participants, along with one caregiver, were provided with transportation to and from their residence and boarding and lodging in a hotel within a block from the laboratory. This was done to minimize travel-related fatigue. Day 1 was intake, and days 2 and 3 were testing days. During intake, participants with PD were consented while on-medication. We then administered the Montreal Cognitive Assessment [MoCA (50)] and recorded a brief history which included disease duration, medications, and medication dosage. Anti-parkinsonian medications were converted to levodopa equivalent daily dosages (LEDD) (51). Afterwards, parts I, II, and IV of the MDS-UPDRS were administered/completed (52). Finally, participants were acclimatized to the laboratory and practiced the experimental tasks in preparation for testing days 2 and 3. Participants practiced until they completely understood the instructions and were able to perform the tasks as instructed. No data were collected during practice. During days 2 and 3, participants with PD performed the same experimental tasks off- and on-medication (1 condition per day). For the off-medication condition, participants refrained from anti-PD medications at least 12 hours prior to testing (53). For the on-medication condition, participants took medication in accordance with their medication schedule. We randomized the order of testing conditions (off- and on-medication) for all participants with PD. On each testing day, testing commenced at 9 am and ended no later than 1 pm. Each day began with the administration of both the Hoehn & Yahr Rating and MDS-UPDRS III (54) followed by the practice and execution of 6 different experimental tasks. Breaks and light snacks were provided between each experimental task. The 2 memory-guided sequential reach tasks were the final tasks executed each day, and the order of these 2 tasks was randomized. Only the findings from the memory-guided sequential reaching tasks will be reported in this paper.

Post-STN-DBS, data collection for participants with PD took place over 5 days. All participants had bilateral implants, and stimulation parameters had been programmed for optimal clinical benefit. Surgical procedures are detailed in David et al. (55). The same procedures were followed at both sites except that at Rush University both leads were implanted on the same day while at Northwestern left and right leads were implanted 6 weeks apart. Day 1 was intake, and days 2–5 were testing days. The intake process was identical to pre-surgery, except that the participants with PD were both on-stimulation and on-medication. During days 2–5, participants performed the same experimental tasks under 4 different conditions (one condition per day): off-bilateral stimulation, on-bilateral stimulation, on-left stimulation, and on-right stimulation. For all conditions, participants refrained from PD medications at least 12 h prior to testing. The order of the testing conditions (off-bilateral, on-bilateral, on-left, and on-right) was randomized for all participants with PD. On each testing day, the experimenter arrived at the participant's hotel room at 6 a.m. to set their stimulation condition for the day. Testing commenced at 9 a.m. and ended no later than 1 p.m. Thus, there was at least a 3-h wash-out period prior to testing. The schedule during testing days was identical to the pre-STN-DBS testing schedule. Only the findings from the memory-guided sequential reaching tasks under the off-bilateral and on-bilateral conditions (henceforth simply referred to as off-STN-DBS and on-STN-DBS) will be reported here. HC performed intake and experimental testing in 1 day.

### Instrumentation

The participants performed the experimental tasks in a completely darkened room to prevent environmental visual cues from aiding target location encoding. The participants were seated upright in an adjustable chair with their chin on a chin-rest to minimize head movements. Consequently, vestibular contributions to proprioception were minimized. Head and finger movements were captured with a 3-D motion capture system (Northern Digital, Waterloo, Canada). Participants had infrared emitting diodes taped to the head-mount of the eye-tracking system and to the right index finger, to track head and finger movements, respectively. For the sequential reaching task encoded with peripheral-vision, eye movements were captured with a head-mounted video-based eye-tracking system, Eyelink II (SR Research, Ottawa, Canada). This data was only utilized to assess proper task performance and was not analyzed further. For the proprioception sequential reach task, the participants were blindfolded, and no eye movements were captured.

A light-emitting diode (LED) (3 mm green LED, 70 mcd), mounted on a central fixation stand (central fixation LED), was situated 42 cm in front of the participant's chin-rest and served as the fixation point for the participant's eye position during the peripheral-vision encoding. A centimeter below this central fixation LED was a finger support, which served as the starting point for the participant's right index finger during both tasks. A second LED (3 mm green LED, 70 mcd) was attached to the arm of a programmable 5-degree-of-freedom (DOF) robot (CRS Robotics Corporation, Burlington, Canada). Using this robot, sequential targets were presented to the participants in a plane that was 42 cm in front of the chin-rest.

For peripheral-vision encoding, the sampling frequency of the eye movements (500 Hz) was down-sampled to the sampling frequency of the head, finger, and robot movements (240 Hz). Eye, head, finger, and robot movements were synchronized and stored using the Motion Monitor system (Innovative Sports Training, Chicago, USA).

### Protocol

#### Peripheral-vision encoding

**Figure 1:**
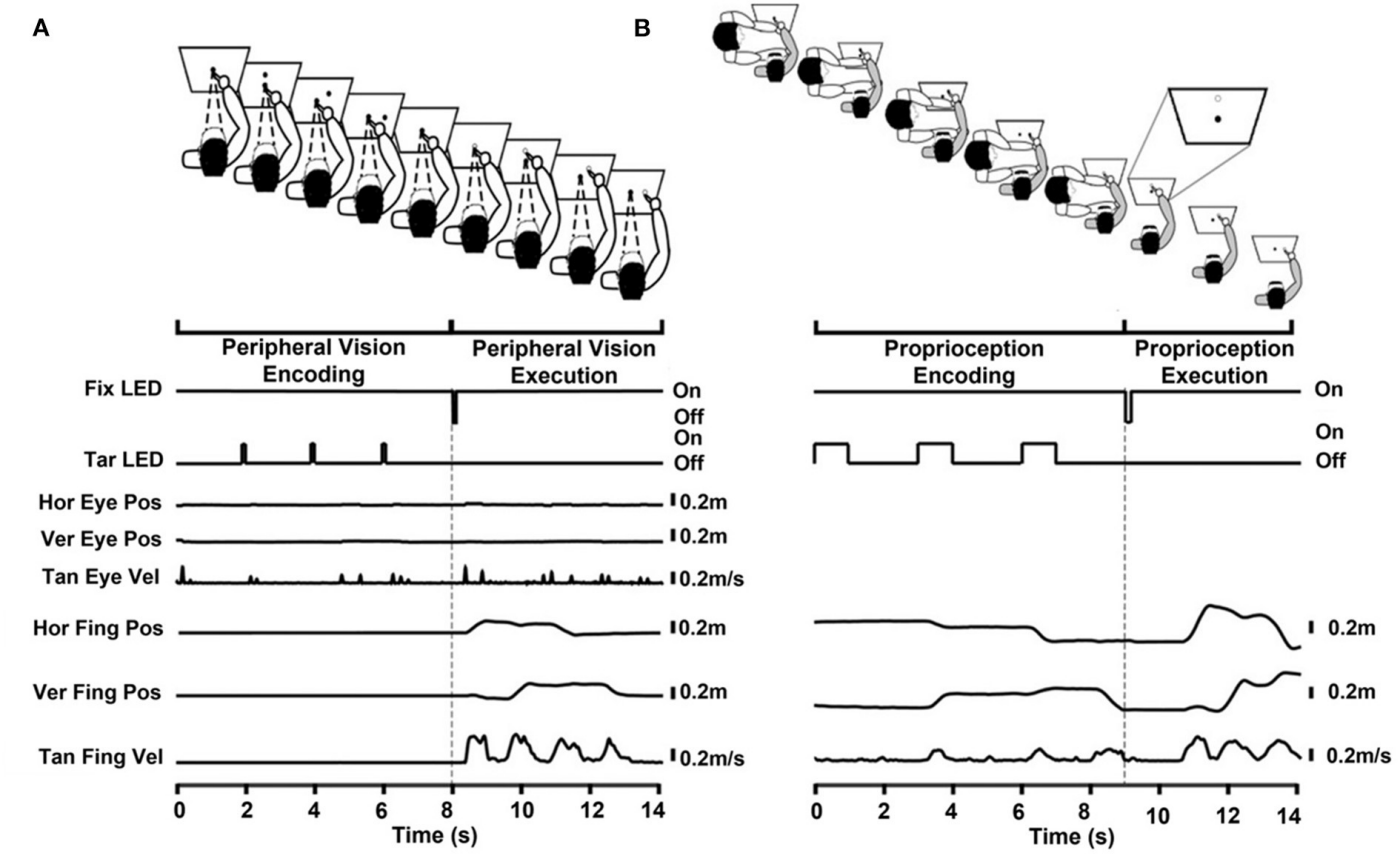
**(A)** Peripheral-visual encoding task divided into the encoding and execution phases. Encoding phase: The participant fixated on the central fixation LED (solid central circle) while placing their right index finger on a stand immediately below the central fixation LED. The target LED (solid peripheral circle) flashed in 3 different locations sequentially. The participant encoded the target location and sequence with their peripheral vision. Execution phase: The flashing of the central fixation LED cued the start of the execution phase (unfilled central circle). The participant remained fixated on the central fixation LED. The participant pointed to the remembered targets (unfilled circles) as accurately as possible in the order presented. The time series below the cartoon show the central fixation LED (Fix LED), target LED (Tar LED), horizontal and vertical eye position (Hor Eye Pos, Ver Eye Pos, respectively), tangential eye velocity (Tan Eye Vel), horizontal and vertical finger position (Hor Fing Pos, Ver Fing Pos, respectively), and tangential finger velocity (Tan Fing Vel). Figures are aligned to the execution cue at 8 s. **(B)** Proprioceptive encoding task divided into the encoding and execution phases. The participant (gray shirt) was blindfolded for the entire task therefor no eye movement traces are shown. Encoding phase: The experimenter (in white) held the participant's (in gray) relaxed right arm at the elbow and wrist while placing their right index finger on a stand immediately below the central fixation LED (solid central circle). The experimenter guided the participant's arm to each of the three sequential targets (solid peripheral circles) ensuring that the participant's pointer finger touched the target LED. The participant encoded the target location and sequence proprioceptively. The experimenter then guided the participant's index finger back to the stand and the participant regained active control of their limb. Execution phase: An oral cue from the experimenter initiated the execution phase. The participant pointed to the remembered targets (unfilled circles, see inset) as accurately as possible in the order presented. The time series below the cartoon show the central fixation LED (Fix LED), the target LED (Tar LED), the horizontal finger position (Hor Fing Pos), the vertical finger position (Ver Fing Pos), and tangential finger velocities (Tan Fing Vel). Figures are aligned to the execution cue at 9 s. Reaching primarily occurred in the horizontal and vertical dimension; therefore, only these traces are shown.

Peripheral-vision encoding began with participants fixating on the central fixation LED for 2,000 ms (0° visual angle) and with their finger resting on the fixation stand. During the encoding phase, the robotic arm flashed the three sequential targets with the central fixation LED still lit. The participants were asked to keep their eyes fixated on the central fixation LED while using their peripheral-vision to encode the location and the sequence of the three targets. The duration of each target presentation was 100 ms, and the time between the onsets of each target presentation was 2,000 ms. The three targets were located on a circular plane with a 10 cm radius. Relative to the fixation LED, the horizontal target was located 0° to the right, the diagonal target was located 45° to the right, and the vertical target was located directly above the central fixation LED at 90°. Targets were presented in random order. After the third target presentation, a final 2,000 ms delay occurred, in which participants had to hold all three targets in memory. The central fixation LED was then extinguished for 100 ms and then lit again. This cue signaled the initiation of the execution phase, during which participants were asked to reach to the remembered sequential targets in the order presented. The central fixation LED stayed on for 5,000 ms, as the participants executed reaching movements to the three memorized targets as accurately as possible, all while keeping their eyes fixated on the central fixation LED. When the reaching movements were completed, participants returned their finger to the central fixation stand. Prior to performing the task, the following task instructions were read to each participant: “*Please fixate on the central fixation LED. While you look at the central fixation LED, 3 targets will flash 1 at a time in your peripheral field of vision. Continue looking at the central fixation LED. After the third target flashes, the LED on the stand will flash. At this time, continue looking at the central fixation LED and point to each target as accurately as you can in the order they appeared. Please hold your finger for an instant at each target location. After pointing to the last target, return your finger to the LED on the stand. You should continue to look at the light on the stand throughout the task.”* Figure 1A illustrates the experimental task along with LED, robot, eye, and finger traces. After every three trials, participants were given a 25 second break. During each break, a flashlight was turned on, and the participant rested their arm on an arm rest. After nine trials, the lights were turned on for 25 s to minimize eye adaptation to the dark.

#### Proprioception encoding

Participants performed the proprioception encoding task with a blindfold on. The task began with the experimenter (FJD) supporting the participant's relaxed right arm at the elbow and wrist. The participant's index finger rested on the fixation stand. During the encoding phase, the experimenter moved the participant's arm to the three sequential targets. The tip of the participant's index finger touched each target. This facilitated proprioception encoding of the target locations and their presentation sequence. Then, the participant's arm was brought back to the fixation stand, and they gained active control over their arm. After a 2,000 ms delay, indicated by the flashing of the central fixation LED, the experimenter orally cued the participant to “go,” initiating the execution phase. Upon hearing the oral cue, the participant reached to the memorized targets as accurately as possible in the sequence that they were presented. When the reaching movement was completed, the experimenter guided the participant's finger back to the central fixation stand in preparation for the next trial. Prior to performing the task, the following instructions were read to each participant: “*FJD will support your elbow and hand from the central stand and move your finger to three targets, 1 at a time. At each location your finger will touch the target. FJD will then bring your arm back to the central stand and let go of your elbow and hand. When FJD says ‘Go,' please point to the targets that you felt, as accurately as you can in the order that they were presented.”* Figure 1B depicts the experimental task along with LED, robot, and finger traces. All three sequential targets were presented with the robotic arm, similar to the peripheral-vision encoding task. The only difference was that the duration of each target presentation was 1000 ms, instead of 100 ms, to provide FJD with enough time to bring the participant's finger to each target. Targets were presented in a random order. Even though targets were presented for a longer duration during proprioception encoding compared to peripheral-vision encoding, the duration that the participant touched the target was brief. In addition, because the pace at which the experimenter moved the participant's limb could influence the participant's reaching velocity, specific instruction were given to reach at similar speeds during both encoding types.

For both encoding types, participants conducted as many practice trials as needed to perform the task satisfactorily. This was followed by 15 test trials. Participants performed 1 block of 15 test trials for each of the medication and stimulation conditions. The randomization sequence within a trial was maintained between blocks of trials; this meant that the target sequence for the 1st trial, 2nd trial, and so on was identical between blocks.

### Data processing

Eye and finger movement data were analyzed using a custom MATLAB script^1^. A 20 Hz low-pass second-order, zero phase Butterworth filter was applied to the eye and finger position signals. The filtered position data were then differentiated to calculate velocity. During the peripheral-vision encoding task, eye position and velocity signals for each trial were visually examined for reflexive eye movement errors. Reflexive eye movement errors were those trials during which an eye movement occurred within 500 ms of target onset and was >50% of the target amplitude. These trials were excluded from further analysis.

For both tasks, processing of the execution phase data started with visually determining the three finger endpoints of each trial on a 2-dimensional representation of the finger movement, which itself was overlaid on a 2-dimensional representation of the target locations and the presentation sequence. This approach was used because most of the change in arm position occurred on the horizontal and vertical planes. The visually determined finger endpoints were then projected onto a time series plot of the tangential finger velocity. The data analyst adjusted the finger endpoints to accurately reflect the offset of each reaching phase, which corresponded to a valley in the tangential velocity profile. The reaching task comprised of four reaching phases. The first, second, and third reaching phases represented reaching to the first, second, and third targets, respectively. The fourth reaching phase occurred when the finger returned to the fixation stand and was not analyzed. The offset of a reach phase served as the onset of the next reach in the sequence. Within each reaching phase, the maximum velocity was defined as the peak velocity. The reaching onset of the first reach phase was computed using its velocity profile. From the velocity peak of the first reaching phase, the algorithm searched backwards to detect the first time point when reaching velocity went below 5% of the peak velocity (56). This time point was defined as the onset of the first reach. Using the location of the reaching endpoints, the reaching amplitude was calculated for each reaching phase. The amplitude of each reach was the magnitude of the vector connecting the start-point and the end-point of each reach. The end-points of the 1st and 2nd reaches served as the start-points for the 2nd and 3rd reaches, respectively. It should be noted that each of the 3 reaches within a trial was of a slightly different amplitude. This is because the reaching amplitude was dependent on the target sequence within a trial. The 1st reach within a trial was always 0.1 m, however the amplitude for the 2nd reach could vary depending on where the 2nd target was located. For example, if the 1st target was directly above the central fixation stand as depicted in Figure 1A, the location of the 2nd target could be in either the diagonal or horizontal location, relative to the central fixation stand. If the 2nd target was in the diagonal location, then the amplitude would have been 0.097 m; whereas if the 2nd target was in the horizontal location, then the amplitude would have been 0.14 m. The average amplitude across all possible reaching sequences was ~0.11 m. Finally, reaching error was calculated by subtracting the values of the finger end point locations from the values of the corresponding target locations. This difference was calculated in the vertical, horizontal, and depth dimensions for each reaching phase endpoint. The magnitude of each reaching endpoint error was subsequently computed using the following equation:


$$\begin{array}{l} \text{Reaching error magnitude}=\sqrt{{{\left(target\;x-endpoint\;x\right)}^{2}{+\left(target\;y-endpoint\;y\right)}^{2}+\left(target\;z-endpoint\;z\right)}^{2}} \end{array}$$

The data for the proprioception encoding task was processed in an identical fashion to the peripheral-vision encoding task with the only difference being that there were no eye traces because the participant was blindfolded.

Individual trial exclusion criteria were as follows: reaching reaction times <0.2 s were excluded as anticipatory reaches and those >1.5 s were excluded as they were deemed trials where the participant was not attending to the instructional set; peak velocities >2 m/s were excluded based on a visual analysis of the frequency distribution; and participants with <3 trials (for encoding type, or medication condition, or stimulation conditions) were not included in the analysis due to insufficient data. Overall, we collected data from 3780 reaches across encoding types, groups, and treatment conditions; of which 373 reaches were excluded. Thus, <10% of reaches were excluded. The number of reaches excluded did not vary as a function of encoding type, medication condition, and STN-DBS condition.

In summary, the following outcomes were included for statistical analysis for each reach within a trial: (1) reaching amplitude in meters, (2) peak reaching velocity in meters/second; and (3) magnitude of reaching error, relative to the target location, in meters.

### Statistical analysis

Descriptive: Each reaching outcome (amplitude, peak velocity, and error) is described using box plots overlaid on violin plots. The box plot provides 4 main features of our data: center (mean and median), spread, asymmetry, and outliers. The violin plot is a smoothened and symmetric kernel density estimate of the frequency for a given value of our outcome variable. The widest regions of the violin plot correspond to the highest density of data. The upper and lower tips correspond to maximum and minimum values of data. The violin plot adds information to the box plot and allows for a quick and meaningful descriptive comparison of distributions of our data between encoding types, groups, and treatment conditions.

Inferential: Each reaching outcome (amplitude, peak velocity, and error) and the MDS-UPDRS III was subject to a mixed-effect regression model. The fixed effects varied depending on the question evaluated and are listed in Table 1. The random effect was the participant. This allowed for the distinction between the within-participant variance and the between-participant variance, thus accounting for correlation within a participant. We also assumed an unstructured correlation structure. Reaching amplitude was included as a covariate for all models assessing reaching velocity because of the well-known association between reaching amplitude and reaching velocity (57). Reaching velocity was included as a covariate for all models assessing reaching error because of the well-known association between reaching velocity and reaching error (36, 57–59). The reported estimated means for velocity and error were adjusted for amplitude and velocity, respectively. In the event of a significant interaction, only the simple main effects are reported in the results section. All statistical analyses used a two-sided 5% level of significance, and *p*-values for *post-hoc* comparisons were adjusted using the Tukey-Kramer method. Normal theory methods and residual diagnostics were used to evaluate validity of assumptions. All statistical analyses were performed using SAS^®^ (version 9.4; SAS Institute, Cary, NC). Our main analysis was a completer analysis. We also conducted an additional analysis with all available data and treated missing data as missing at random.

**Table 1:** Fixed effects used in the mixed models used.

**Question evaluated**	**Fixed effects**	**Outcomes**
1. The effect of encoding and group	Encoding^*a*^, Group^*b*^, Encoding by Group interaction	Amplitude
2. The effect of medication	Encoding^*a*^, Treatment^*c*^, Encoding by Treatment interaction	Velocity
3. The effect of STN-DBS in PD	Encoding^*a*^, Treatment^*d*^, Encoding by Treatment interaction	Error
4. a) The effect of on STN-DBS and on meds	Encoding^*a*^, Treatment^*e*^, Encoding by Treatment interaction	
b) The effect of STN-DBS and meds	Modality^*f*^, Treatment^*g*^, Modality by Treatment interaction	MDS-UPDRS III

HC, healthy controls; PD, Parkinson's disease; STN-DBS, subthalamic nucleus deep brain stimulation; MDS-UPDRS III, Movement Disorders Society Unified Parkinson's Disease Rating Scale Motor Score.

^*a*^Encoding: peripheral vision and proprioception.

^*b*^Group: HC and PD (pre-surgery, while off medication).

^*c*^Treatment was medication: off and on medication (pre-surgery).

^*d*^Treatment was STN-DBS: off and on STN-DBS (post-surgery, while off medication).

^*e*^Treatment: on STN-DBS (post-surgery) and on medication (pre-surgery).

^*f*^Modality: STN-DBS and medication.

^*g*^Treatment: on and off.

## Results

**Figure 2:**
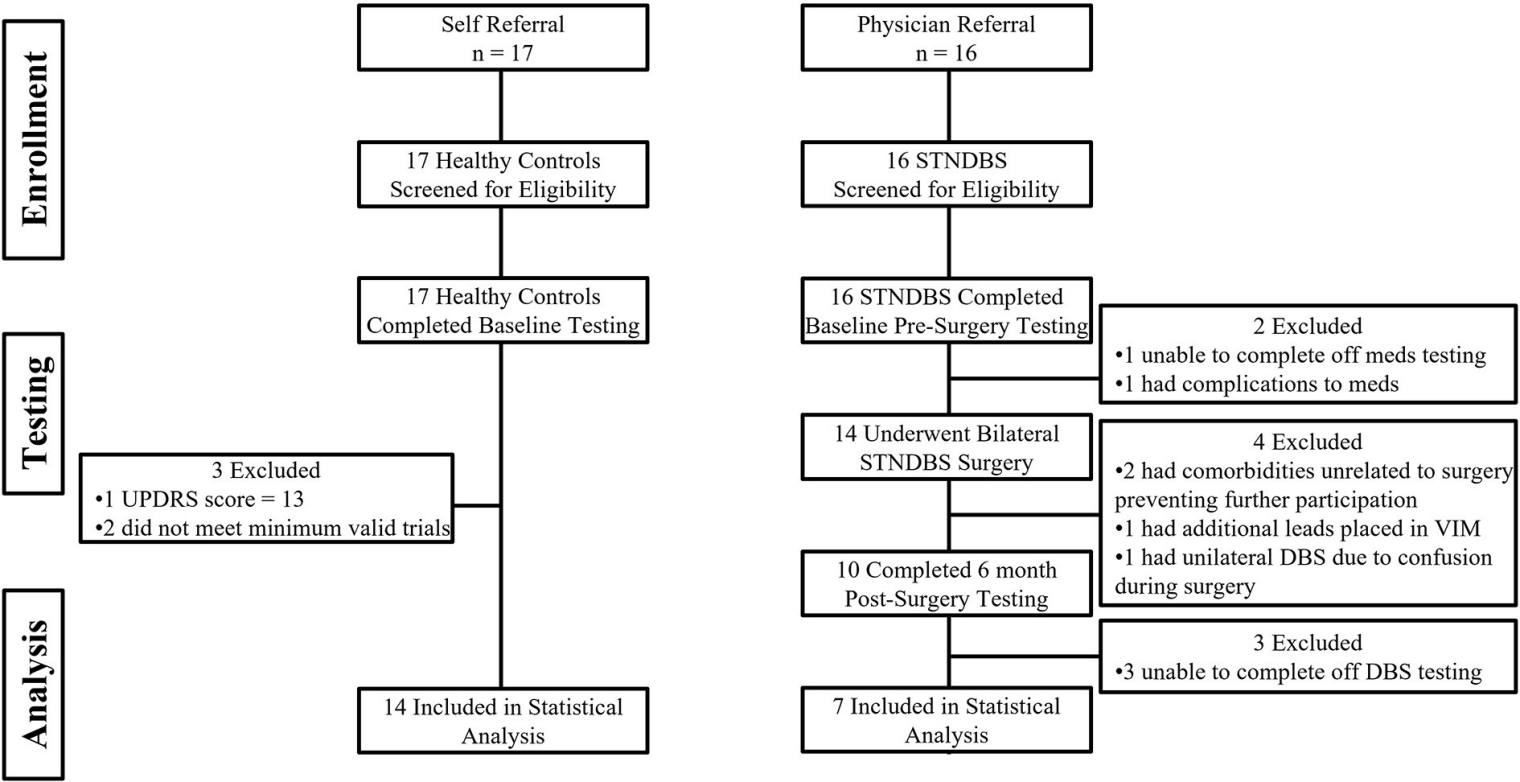
Shows the participant flow in the healthy control group and the Parkinson's disease group.

Of the 17 HC participants, three were excluded from further analysis. One had an MDS-UPDRS III score of 13 points. The other two had an insufficient number of valid trials to be included in the analysis. Of the 16 participants with PD, nine participants were excluded from the completer analysis. Pre-surgery, one participant was unable to go off-medication, and 1 was unable to go on-medication due to severe side effects. Post-surgery, four participants were lost to follow-up: two participants had comorbidities unrelated to the surgery that prevented further participation, one participant had additional leads placed in the ventral intermediate nucleus of the thalamus due to the inability to stop severe tremors without inducing dyskinesias and speech impairment, and one participant became confused during surgery and a DBS lead was implanted only unilaterally. In addition, three were unable to go off-stimulation following surgery. Figure 2 highlights the flow of participants in both groups. Supplementary Table 1 compares the demographic and clinical data between completers and non-completers. The completers and non-completers were similar with respect to age, disease duration, MoCA, and off MDS-UPDRS III scores. The completers had a LEDD that was 415 mg greater than non-completers; however, this was not statistically significant. Supplementary Table 2 compares the findings from the completer analysis (PD: *n* = 7; HC: *n* = 14) with the additional analysis that was performed with all available data which treated missing data as missing at random (PD: *n* = 15; HC: *n* = 14). The results and conclusions from these two analyses were similar. Here, we report findings from the completer analyses, i.e., from those who had a complete set of data on both experimental tasks, treatment conditions, and time points (PD: *n* = 7; HC: *n* = 14). Tables 2, 3 provide a summary of group demographics and individual participant demographics and stimulation settings for completers, respectively.

**Table 2:** Demographic table (mean ± standard deviation).

	**HC**	**Pre STN-DBS surgery**	**Post STN-DBS surgery**
Sex (M/F)	12/2	7/0	7/0
Age (years)	65.43 ± 4.24	65.86 ± 3.89	66.71 ± 3.64
Disease duration (years)	N/A	10.71 ± 5.47	11.71 ± 5.47
Months post-surgery	N/A	N/A	7.86 ± 1.46
MOCA	27.21 ± 1.63	27.86 ± 1.95	26.71 ± 2.21
OFF MDS-UPDRS III	2.93 ± 2.30	48.71 ± 10.40^*a*^	53.29 ± 15.38^*b*^
ON MDS-UPDRS III	N/A	39.29 ± 12.47^*c*^	18.29 ± 7.65^*d*^
LEDD (mg)	N/A	1319.29 ± 817.95	449.29 ± 246.58

HC, Healthy controls; STN-DBS, Subthalamic nucleus deep brain stimulation; M/F, Male/Female (count); MOCA, Montreal Cognitive Assessment; MDS-UPDRS III, Movement Disorder Society Unified Parkinson's Disease Rating Scale Motor Score; LEDD, Levodopa Equivalent Daily Dose; mg, milligrams.

^*a*^Off medication (overnight withdrawal from medication).

^*b*^Off stimulation (3-hour washout) and off medication (overnight withdrawal from medication).

^*c*^On medication.

^*d*^On stimulation and off medication (overnight withdrawal from medication).

**Table 3:** Participant demographics and stimulation settings.

**ID**	**Sex**	**Age**	**Disease duration (years)**	**Months post surgery**	**MoCA**	**MDS-UPDRS III**		**Left stimulator settings**					**Right stimulator settings**				
						**OFF^1^**	**ON^2^**	**Amplitude (V or mA)**	**Frequency (Hz)**	**Pulse width (μsec)**	**Contact**		**Amplitude (V or mA)**	**Frequency (Hz)**	**Pulse width (μsec)**	**Contact**	
											**+**	**-**				**+**	**-**
1^M^	M	61	17	6	30	43	12	3	130	60	1	0	4	130	60	10	9
3^M^	M	66	8	8	27	61	10	3	125	60	1	2	3.2	125	60	Case	9
8^A^	M	64	6	10	25	44	21	2.4	130	60	Case	3abc	3.2	130	90	Case	10abc
12^A^	M	72	14	6	27	70	14	2.9	130	60	Case	3abc	2.9	130	60	Case	10abc
13^A^	M	67	5	8	28	28	15	3	130	60	Case	2abc	3.1	130	60	Case	10abc
14^A^	M	70	19	8	27	59	25	2.1	130	60	Case	2c	2.7	130	60	Case	10c
17^A^	M	67	13	9	23	68	31	3.6	130	60	Case	2c	2.5	180	60	Case	10abc

MOCA, Montreal Cognitive Assessment; MDS-UPDRS III, Movement Disorder Society Unified Parkinson's Disease Rating Scale Motor Score; V, Volts; mA, milli Ampere; Hz, Hertz; μsec, microseconds; M, Male; abc, tripartite segmental active contacts for flexibility in programming.

^1^Off stimulation (3-hour wash-out from stimulation and overnight withdrawal from medication, post-surgery).

^2^On stimulation and off medication (overnight withdrawal from medication, post-surgery).

^M^Medtronic 3389 (manufacturer and lead model implanted in participant).

^A^Abbott 6172 (manufacturer and lead model implanted in participant).

**Figure 3:**
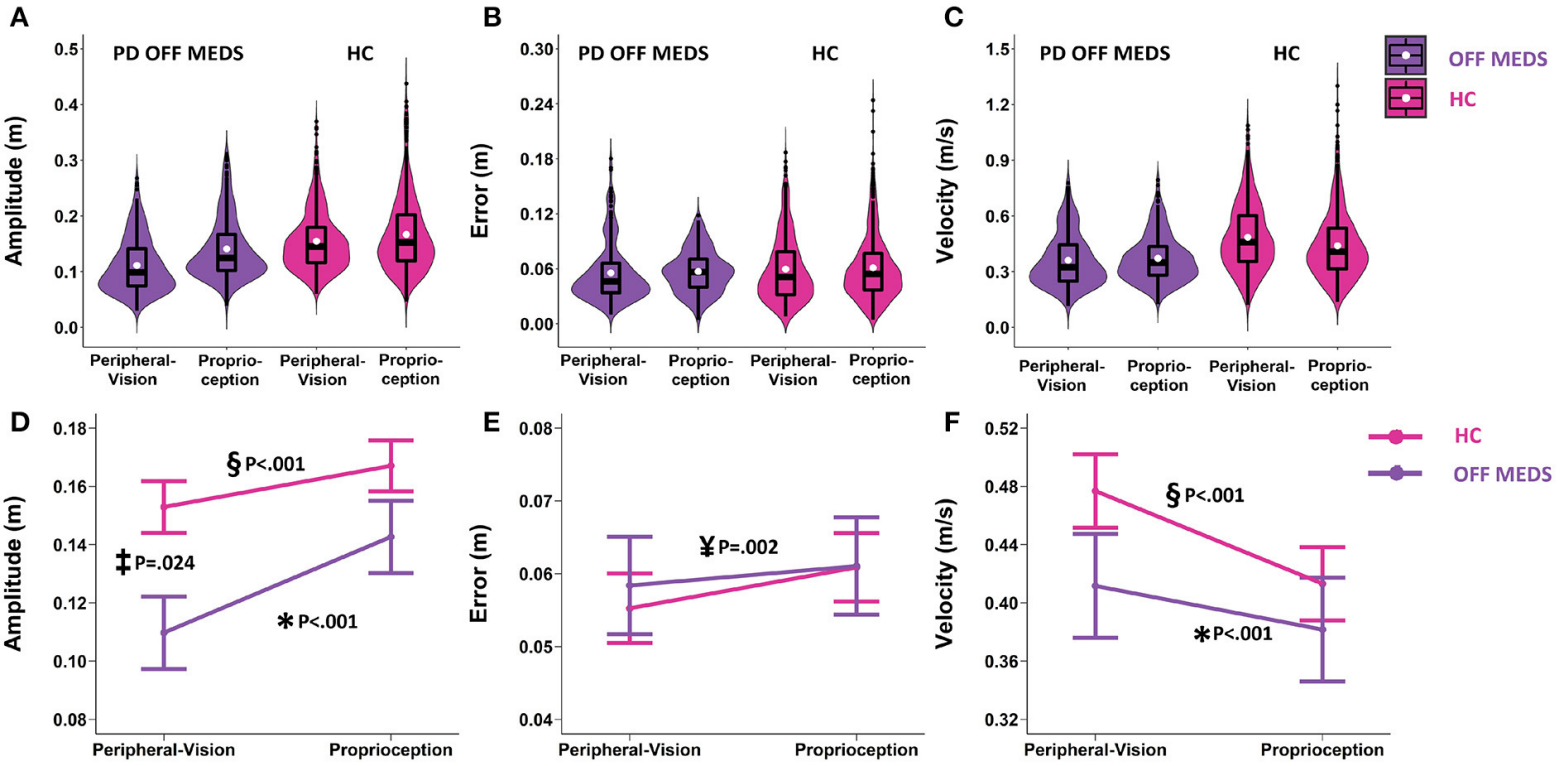
Top row: Box plots overlaid with violin plots of observed reaching amplitude **(A)**, error **(B)**, and velocity **(C)** for PD OFF Medication (OFF MEDS, purple) and Healthy Controls (HC, pink). The boxplot shows the 25th, 50th, and 75th percentiles (horizontal black lines), filled white circle represents the mean, and filled black circles are outliers. Bottom row: Linear mixed model estimated mean ± SE of reaching amplitude **(D)**, error **(E)**, and velocity **(F)** for PD OFF Medication (OFF MEDS, purple) and Healthy Controls (HC, pink) for the peripheral-vision encoding and proprioception encoding. **(D)** Asterisk (*) and double-s (§) indicate statistically significant smaller amplitudes during peripheral-vision relative to proprioception encoding for the PD OFF MEDS (purple) and HC (pink) groups respectively. Double dagger (‡) indicates statistically significant lower amplitudes in PD OFF MEDS relative to HC, only during peripheral-vision encoding. **(E)** Yen (¥) indicates statistically significant main effect of encoding type, i.e., averaging across groups, peripheral-vision reaches were lower in error relative to proprioception reaches. **(F)** Asterisk (*) and double-s (§) indicate statistically significant faster velocities during peripheral-vision relative to proprioception encoding in PD OFF MEDS (purple) and HC (pink) groups, respectively. The trends seen in the observed data in the top row are replicated in the estimated means in the bottom row and those differences that are statistically significant are illustrated with symbols.

**Figure 4:**
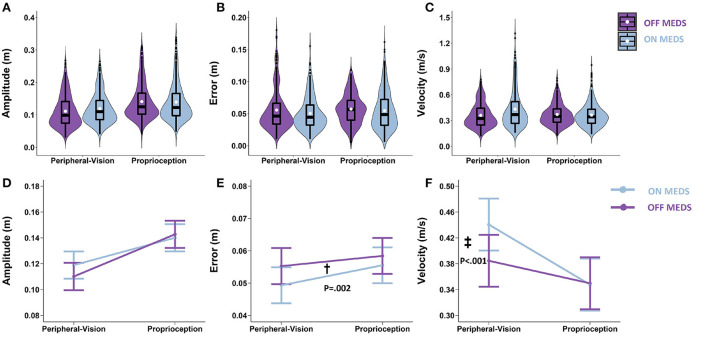
Top row: Box plots overlaid with violin plots of observed reaching amplitude **(A)**, error **(B)**, and velocity **(C)** for PD OFF Medication (OFF MEDS, purple) and PD ON Medication (ON MEDS, blue). The boxplot shows the 25th, 50th, and 75th percentiles (horizontal black lines), filled white circle represents the mean, and filled black circles are outliers. Bottom row: Linear mixed model estimated mean ± SE of reaching amplitude **(D)**, error **(E)**, and velocity **(F)** for PD ON Medication (ON MEDS, blue), and OFF Medication (OFF MEDS, purple) for the peripheral-vision and proprioception encoding. **(D)** Medication had no effect on amplitude for both encoding types. **(E)** Dagger (†) indicates a statistically significant main effect of medication, i.e., averaging across encoding types, ON MEDS reduced error relative to OFF MEDS. **(F)** Double dagger (‡) indicates statistically significant increase in velocity while ON MEDS relative to OFF MEDS, only during peripheral-visual encoding. The trends seen in the observed data in the top row are replicated in the estimated means in the bottom row and those differences that are statistically significant are illustrated with symbols.

**Figure 5:**
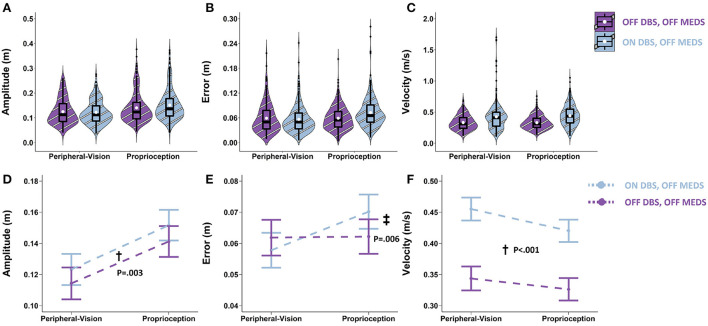
Top row: Box plots overlaid with violin plots of observed reaching amplitude **(A)**, error **(B)**, and velocity **(C)** for PD OFF bilateral STN-DBS (OFF DBS, striped purple) and ON bilateral STN-DBS (ON DBS, striped blue). The boxplot shows the 25th, 50th, and 75th percentiles (horizontal black lines), filled white circle represents the mean, and filled black circles are outliers. Bottom row: Linear mixed model estimated mean ± SE of reaching amplitude **(D)**, error **(E)**, and velocity **(F)** for PD ON bilateral STN-DBS (ON DBS, dashed blue), and OFF bilateral STN-DBS (OFF DBS, dashed purple) for the peripheral-vision and proprioception encoding. All STN-DBS testing was conducted while OFF medication. **(D)** Dagger (†) indicates a statistically significant main effect of STN-DBS, i.e., averaging across encoding types, ON DBS increased amplitude relative to OFF DBS. **(E)** Double dagger (‡) indicates statistically significant increase in error while ON DBS relative to OFF DBS, only during proprioception encoding. **(F)** Dagger (†) indicates a statistically significant main effect of STN-DBS, i.e., averaging across encoding types, ON DBS increased velocity relative to OFF DBS.

**Figure 6:**
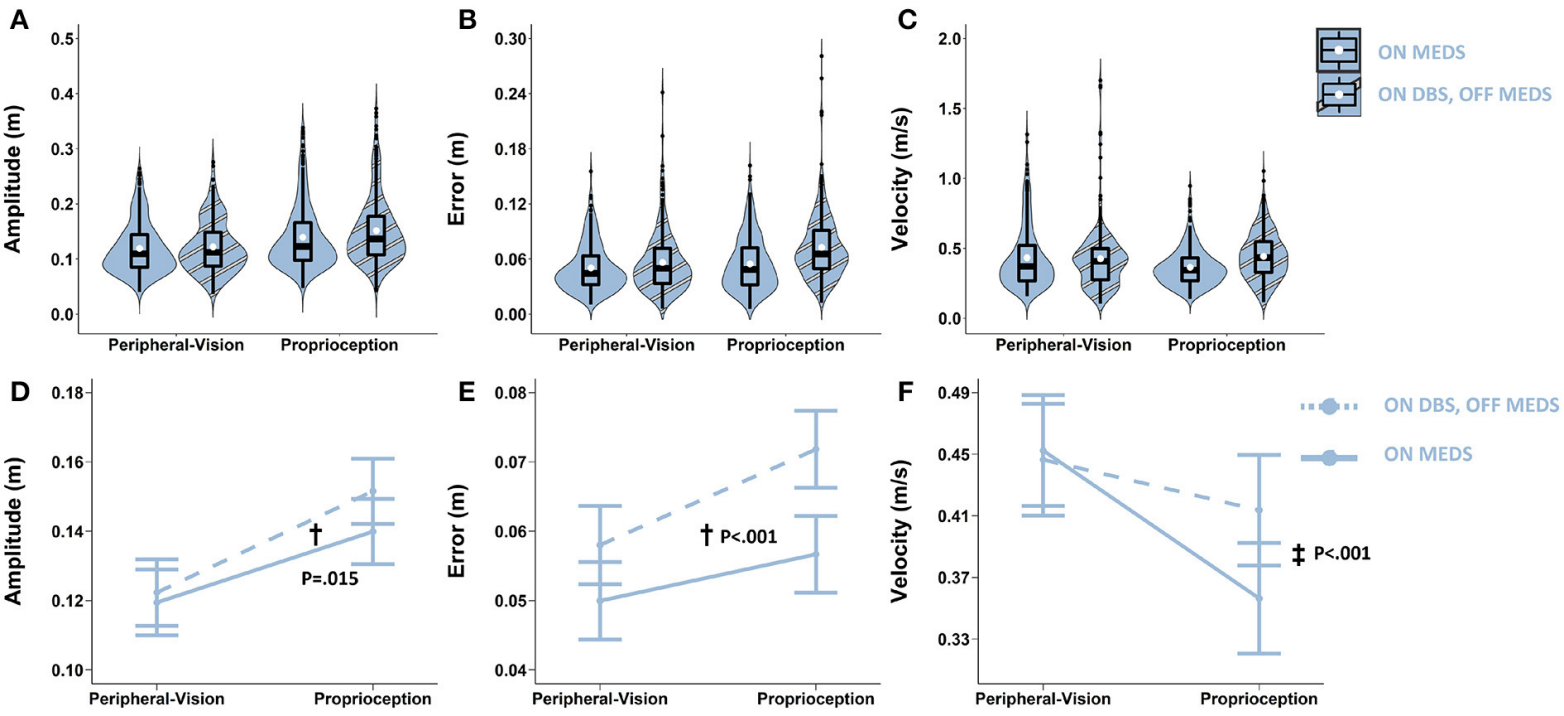
Top row: Box plots overlaid with violin plots of observed reaching amplitude **(A)**, error **(B)**, and velocity **(C)** for PD ON Medication pre-surgery (ON MEDS, blue) and PD ON bilateral STN-DBS post-surgery (ON DBS, striped blue). The boxplot shows the 25th, 50th, and 75th percentiles (horizontal black lines), filled white circle represents the mean, and filled black circles are outliers. Bottom row: Linear mixed model estimated mean ± SE of reaching amplitude **(D)**, error **(E)**, and velocity **(F)** for PD participants ON Medication pre-surgery (solid light blue) and ON bilateral STN-DBS post-surgery (dashed light blue) for the peripheral-vision and proprioception encoding. All STN-DBS testing was conducted while OFF medication. **(D)** Dagger (†) indicates a statistically significant main effect of treatment, i.e., averaging across encoding types, ON DBS increased amplitude relative to ON MEDS. **(E)** Dagger (†) indicates a statistically significant main effect of treatment, i.e., averaging across encoding types, ON DBS increased error relative to ON MEDS. **(F)** Double dagger (‡) indicates statistically significant increase in velocity while ON DBS relative to ON MEDS, only during proprioception encoding.

As a general organizational note, Figures 3–**6** are presented in two rows. The top row (panels A, B, and C in Figures 3–**6**) presents a box plot overlaid on a violin plot (60). The bottom row (panels D, E, and F in Figures 3–**6**) presents the linear mixed model estimated means and their standard errors. The bottom row compliments the top row and makes it clear where statistically significant differences are observed. This presentation format completely describes our data. Note that slight differences in the observed statistics (top row) and estimated statistics (bottom row) are expected. This is because the estimated statistics are linear mixed model based estimates, and in the case of error and velocity, they are adjusted for velocity and amplitude, respectively. The rest of the results section will focus only on the findings from the linear mixed model analyses as the primary focus of this paper is to make inferences from our sample.

### Encoding (peripheral-vision vs. proprioception) and group (PD off medication pre-surgery vs. HC)

Amplitude: We found an encoding type by group interaction (F_1.1634_ = 13.4, *p* < 0.001). The simple main effects of encoding type were similar across PD (off-medication pre-surgery) and HC. In both groups, compared to proprioception encoding, peripheral-visual encoding resulted in reaches that were smaller in amplitude compared to proprioception encoding (PD: smaller by 0.033 m, *p* < 0.001, see Figure 3D data in purple and significance denoted by “^*^”; HC: smaller by 0.014 m, *p* < 0.001, see Figure 3D data in pink and significance denoted by “§”). The simple main effects of group varied as a function of encoding type. PD had significantly smaller reaches than HC during peripheral-vision encoding (smaller by 0.043 m, *p* = 0.024, this comparison is denoted by “‡” in Figure 3D) but not during proprioception encoding (0.024 m, *p* = 0.376).

Error: The encoding type by group interaction was not significant (F_1.1659_ = 1.24; *p* = 0.266, see Figure 3E). We found a main effect of encoding type (F_1.1659_ = 9.84, *p* = 0.002). Averaging across groups, we found that peripheral-vision encoding resulted in reaches that were slightly lower in error compared to proprioception encoding (lower by 0.004 m, *p* = 0.002, see Figure 3E, significance denoted by “¥”). The main effect of group was not significant (F_1.1659_ = 0.04; *p* = 0.838).

Velocity: We found an encoding type by group interaction (F_1.1633_ = 12.51, *p* < 0.001). The simple main effects of encoding type were similar across PD and HC. In both groups, compared to proprioception encoding, peripheral-visual encoding resulted in reaches that were faster compared to proprioception encoding (PD: faster by 0.029 m/s, *p* < 0.001, see Figure 3F data in purple and significance denoted by “^*^”; HC: faster by 0.064 m/s, *p* < 0.001, see Figure 3F data in pink and significance denoted by “§”). The simple main effects of group were not significant. PD had similar reaching velocities to HC during peripheral-vision encoding (0.065 m/s, *p* = 0.442, see Figure 3F) and during proprioception encoding (0.031 m/s, *p* = 0.888, see Figure 3F).

In all subsequent models, the effects of encoding type on amplitude, error, and velocity were similar to what was reported in this section, i.e., peripheral-vision encoding reaches were smaller in amplitude, slightly lower in error, and faster in velocity. To reduce repetition, the main effects of encoding in the absence of an interaction will not be reported in the rest of the results section (61–64).

### Medication: On- vs. off- medication (pre-surgery)

With respect to amplitude, the main effect of medication was not significant (F_1.1126_ = 1.27; *p* = 0.260). Averaging across encoding types, the difference between on- and off- medication was 0.003 m (*p* = 0.260; Figure 4D). With respect to error, the main effect of medication was significant (F_1.1140_ = 9.44; *p* = 0.002). Averaging across encoding types, medication reduced error by 0.004 m (*p* = 0.002; denoted by “†” in Figure 4E). With respect to velocity, the medication by encoding interaction was significant (F_1,1125_ = 28.38; *p* < 0.001). Medication interacted with encoding type and selectively increased velocity only during peripheral-vision encoding by 0.056 m/s (*p* < 0.001; denoted by “‡” in Figure 4F).

### STN-DBS: On- vs. off- STN-DBS (post-surgery, while off- medication)

With respect to amplitude, the main effect of STN-DBS was significant (F_1,990_ = 8.69; *p* = 0.003). Averaging across encoding types, STN-DBS increased amplitude by 0.010 m (*p* = 0.003; denoted by “†” in Figure 5D). With respect to error, the STN-DBS by encoding interaction was significant (F_1.989_ = 10.36; *p* = 0.001). STN-DBS interacted with encoding type and increased error only during proprioception encoding (0.008 m; *p* = 0.006; denoted by “‡” in Figure 5E). With respect to velocity, the main effect of STN-DBS was significant (F_1.989_ = 152.8; *p* < 0.001). Averaging across encoding types, STN-DBS increased velocity by 0.103 m/s (*p* < 0.001; denoted by “†” in Figure 5F).

### STN-DBS (post-surgery) vs. medication (pre-surgery)

#### Reaching outcomes

With respect to amplitude, the main effect of on-treatments was significant (F_1.1096_ = 5.9; *p* = 0.015). Averaging across encoding types, relative to on-medication, on-STN-DBS increased amplitude by 0.007 m (*p* = 0.015; denoted by “†” in Figure 6D). With respect to error, the main effect of on-treatments was significant (F_1.1104_ = 47.21; *p* < 0.001). Averaging across encoding types, relative to on-medication, on-STN-DBS increased error by 0.012 m (*p* < 0.001; denoted by “†” in Figure 6E). With respect to velocity, the on-treatments by encoding interaction was significant (F_1,989_ = 13.9; *p* < 0.001). Relative to on-medication, on-STN-DBS interacted with encoding type and increased velocity only during proprioception encoding by 0.057 m/s (*p* < 0.001; denoted by “‡” in Figure 6F).

#### MDS-UPDRS III

**Figure 7:**
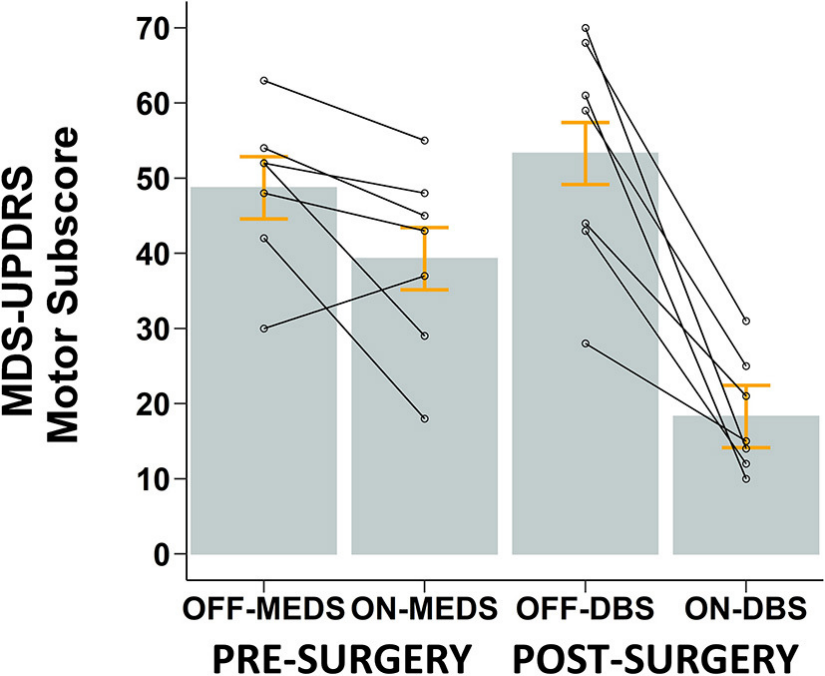
Mean ± SE (gray bars and orange error bars) of MDS-UPDRS Motor Scores for PD participants pre-surgery while OFF and ON anti-parkinsonian medication and post-surgery while OFF and ON STN-DBS. Overlaid open circles and connecting lines show MDS-UPDRS Motor Scores of each participant. All STN-DBS testing was conducted while OFF medication. All post-surgery data was collected following 12-h overnight withdrawal from anti-Parkinsonian medication.

As can be seen in Figure 7, medication reduced the MDS-UPDRS III score in all but one participant. STN-DBS decreased the MDS-UPDRS III score across all participants. Figure 7 shows that, on average, medication reduced the MDS-UPDRS III score by 9.4 points, a 19.3% decrease (*p* = 0.070), while STN-DBS had a dramatic effect and reduced the MDS-UPDRS III score by 35 points, a 65.7% decrease (*p* < 0.001).

## Discussion

We investigated the effect of encoding (peripheral-vision vs. proprioception), the effect of medication (on- vs. off-), the effect of STN-DBS (on- vs. off- STN-DBS while off-medication), and compared the effect of STN-DBS vs. medication on reaching amplitude, error, and velocity during memory-guided sequential reaching in participants with PD. First, we found that peripheral-vision encoding resulted in reaches that were smaller in amplitude, slightly lower in error, and faster than those reaches performed with proprioception encoding. This presentation pattern was similar to HC. However, participants with PD manifested deficits in amplitude compared to HC, and these deficits were larger during peripheral-vision encoding. Second, we found that medication had no effect on amplitude for both encoding types, reduced error for both encoding types, and selectively increased velocity during peripheral-vision encoding. Third, we found that STN-DBS increased amplitude for both encoding types, selectively increased error during proprioception encoding, and increased velocity for both encoding types. Fourth, while comparing STN-DBS to medication we found that STN-DBS was better than medication with respect to increasing amplitude for both encoding types, increasing velocity for proprioception encoding, and reducing motor signs of PD on the MDS-UDPRS III. In contrast, medication was better than STN-DBS with respect to reducing error for both encoding types. We discuss each of our findings below.

### The effect of encoding type

In participants with PD, relative to proprioception encoding, peripheral-vision encoding resulted in reaches with a significantly smaller amplitude and lower error (Figures 3D,E). This presentation pattern was similar to HC evaluated in our study and is consistent with two previous studies (17, 65). In addition, a novel finding in our study is that despite amplitudes being smaller, reaching velocities adjusted for amplitude were larger during peripheral-vision encoding compared to proprioception encoding (Figure 3F). Previous studies (17, 65) have not reported reaching velocities when comparing vision and proprioception encoded reaches. Peripheral-vision encoding appears to offer a benefit over proprioception encoding in that these reaches are faster in velocity despite being smaller in amplitude. During peripheral-visual encoding, the participant can rely on both visual and proprioceptive inputs during execution; however, during the proprioception encoding, the participant can rely on only proprioceptive inputs during execution. It is known that having both visual and proprioceptive inputs enhances limb localization (66). Similarly, having both visual and proprioceptive inputs provide greater certainty about target location as well. It has been shown that when certainty increases, movement duration decreases (67), and consequently movement speed increases. Thus, it is likely that the enhanced limb localization and greater target certainty during execution facilitated more accurate and faster movements during peripheral-vision encoding.

Relative to HC, reaching deficits in participants with PD were consistent with the well-established deficit of hypokinesia, i.e., reduced amplitude (61, 68), but there were no deficits in reaching velocity or error. Of note, the amplitude deficits were observed during peripheral-vision encoding compared to proprioception encoding (Figure 3D). The reason for this is likely because peripheral-vision encoding was cognitively more complex than proprioception encoding. It is known that as the cognitive complexity increases, movement deficits, relative to HC, increase in participants with PD (69). During peripheral-vision encoding the visual system is used to encode target location from an eye-centric frame of reference. This information must be transformed into a limb-centric frame of reference during execution of the reach. Whereas, during proprioception encoding, encoding and execution occur in a limb-centric frame of reference. There is no transfer of information between different sensory systems. Thus, peripheral-vision encoding is more complex than proprioception encoding. This might explain why we observed amplitude deficits that were greater during peripheral-vision compared to proprioception encoded reaching. The lack of a statistical significance with respect to velocity between PD and HC, during peripheral-vision encoding is attributed to the greater variability observed (see error bars in Figure 3F). In addition, we may not have been sufficiently powered to detect differences between groups with respect to velocity.

### The effect of medication

Medication had no effect on the amplitude deficit that was observed during peripheral-vision encoding (Figure 4D); however, medication increased velocity for the peripheral-vision encoding (Figure 4F). This finding is consistent with previous studies that have shown that dopaminergic medication preferentially improves movement velocity but not amplitude (70, 71). Why this is the case remains unknown. One possible explanation for this differential effect can be attributed to emerging evidence which suggests that the output nuclei of the basal ganglia, the globus pallidus and the substantia nigra pars reticulata (72), encode amplitude and position of movement, while the striatal neurons encode velocity (73). In addition, the globus pallidus are sparsely innervated with dopaminergic neurons, while the striatum is densely innervated with dopaminergic neurons (74). Medication is likely to impart its beneficial effects at the striatum, where the dopaminergic loss and denervation is the most in PD (75). This could result in velocity being improved and amplitude being unaffected.

Another noteworthy finding is that medication reduced error during both encoding types (Figure 4E). This finding is consistent with previous reports that have shown that levodopa improves working memory performance on the N-Back task (76, 77). This improvement is thought to be brought about by a levodopa induced increase in resting state functional connectivity between the caudate and parietal cortex, which is part of the fronto-parietal attentional network (77). This fronto-parietal network overlaps with those neural areas involved in spatial location and spatial working memory (16). In addition, these areas have also been shown to be engaged during proprioceptive reaches (18). Therefore, there is a biological basis for a medication induced reduction in spatial error during both peripheral-vision and proprioception encoded memory-guided reaching.

### The effect of STN-DBS

STN-DBS improved amplitude for both encoding types, selectively worsened error during proprioception encoding, and improved velocity for both encoding types (Figure 5). Our findings were consistent with previous studies that showed that STN-DBS improved intensive aspects of control such as movement amplitude and velocity but impaired coordinative/integrative aspects of control such as error (36, 43, 78, 79). Of note is the amount that STN-DBS increased error: it was 0.008 m. While this was statistically significant, the clinical significance of this error magnitude may not be readily apparent. The average amplitude of an accurate reach was about 0.1 m. So, an error of 0.008 m is 8% of the amplitude of the reach. In relative terms, this is an error magnitude that is 8% of the reach amplitude. This is quite significant. In addition, when a participant with PD enters a novel environment, it is impossible to foveate all available handholds that a participant with PD can reach out for in the event of a trip or loss of balance. Reliance on peripheral-vision is inevitable. However, participants with PD are known to have peripheral-visual deficits (8, 9), in addition, they have proprioceptive deficits (10, 11), and are hypokinetic and bradykinetic (61, 68). Taken together, all of these deficits are likely to magnify an 8% error in reaching and make it clinically significant in a non-lab environment. Furthermore, it does not matter if one misses a reach to a memorized handhold by a few millimeters or a few centimeters; a miss is a miss, and the result is a serious adverse event for a participant with PD. Thus, an increase in error of 0.008 m is both statistically and clinically significant.

High-frequency STN-DBS modulates oscillatory activity in the basal ganglia-thalamo-cortical circuit (80). Specifically, it attenuates beta band activity, facilitates beta power desynchronization, enhances gamma power synchronization, and reduces the phase amplitude coupling between beta and gamma oscillations, which is associated with clinical improvements (81–84). This STN-DBS induced suppression of low frequency and enhancement of high frequency oscillatory patterns forms the basis for improving intensive aspects of amplitude and velocity. That being said, coupling between low and high frequency oscillations has been shown to be associated with memory and sensory-motor integration (85). Memory and sensory-motor integration are critical for coordinative aspects of movement because they are required to synthesize information about amplitude, velocity, and location in order to plan and execute accurate movements. It is theorized that STN-DBS might disrupt power in low frequency bands, such as theta and alpha, and reduce coupling between low and high frequencies that underlie the integrative aspects of movement (36, 43, 78). This disruption could impair sensory-motor integration and may have driven the greater error observed during proprioceptive reaching (Figure 5E). However, it should be noted that error was not affected during peripheral-vision encoding. It is not clear why error was increased only during proprioception encoding and not during peripheral-vision encoding. Perhaps the presence of peripheral-vision during encoding could compensate for the STN-DBS induced disruption of low frequency oscillations. This idea requires further evaluation.

### STN-DBS vs. medication

The findings from the comparison of STN-DBS with medication were complicated and were dependent on encoding type and reaching outcome. STN-DBS was better than medication at increasing amplitude (Figure 6D) and velocity (Figure 6F) for proprioceptive encoding but was similar to medication at increasing velocity for peripheral-vision encoding (Figure 6F). In addition, STN-DBS was better than medication in reducing motor signs of PD as quantified by the MDS-UDPRS III (Figure 7). In contrast, medication was better than STN-DBS in reducing error for both encoding types (Figure 6E). Taken together, these findings support the idea that STN-DBS is superior to medication with respect to some intensive aspects of movement and medication is superior to STN-DBS with respect to coordinative aspects of movement. It also supports the idea that while STN-DBS and medication share overlapping mechanisms of action, there are likely unique mechanisms of action at play. For instance, overlapping mechanisms include the fact that both levodopa and STN-DBS suppress beta power and reduce beta power synchronization in the basal ganglia-thalamo-cortical network, and these effects are linked with the clinical benefit of both treatments (78). In addition, following STN-DBS surgery, there is a significant reduction in LEDD (Table 2) which also suggests overlapping mechanisms are at play (86). On the other hand, unique mechanisms include the fact that levodopa acts primarily on the striatum and can affect the direct and indirect pathway (87), while STN-DBS acts on the STN and primarily affects the indirect pathway (88). Another line of evidence is that, in humans, most studies indicate that STN-DBS does not increase striatal dopamine levels (89–92), even though, in theory, this may be possible (93) and has been shown in one study (94). Finally, non-dopaminergic mechanisms are unaffected by dopaminergic medication but can be altered by STN-DBS. For instance, the sequence effect, the progressive decrement in movement amplitude with repetitive movement, does not respond to dopaminergic medication (95–97) but has been shown to improve with STN-DBS (98). It should be noted that our sample of participants with PD were going to undergo STN-DBS surgery; as such they had a physician documented positive response to a supra-threshold dose of levodopa but were fluctuators with regards to their response to levodopa.

Of note is the dramatic effect STN-DBS had on the MDS-UPDRS III compared to the effect medication had on the MDS-UPDRS III (Figure 7). This effect was substantially larger than previously published data (99). One possible reason for this is that Weaver and colleagues (99) used standard DBS leads, whereas in our study, five out of seven participants had newer DBS leads which had two levels of tripartite electrodes. These leads have been shown to provide a larger therapeutic window (100), with one study reporting a 60% improvement in the MDS-UPDRS III score for the on-STN-DBS condition compared to off-STN-DBS (101). In addition, a more recent study also reported a 60% improvement on the UPDRS-III (102). This 60% improvement while on-STN-DBS observed in more recent studies is consistent with the improvement seen in our study (101, 102). Moreover, recent technological advances in the design of DBS leads and implantable pulse generators have increased flexibility for programming that may have led to the dramatic effect of STN-DBS observed in our study (103).

Finally, the effect of medication was not statistically significant on the MDS-UPDRS III. However, the reduction in MDS-UPDRS III exceeds the minimally clinically important difference of 3.25 points (104). This effect of medication on the MDS-UPDRS III in our sample is not as large as previous reports, possibly due to the following three reasons. First, we had a lower LEDD in our sample compared to other samples [ours: 1,144 mg; Weaver et al.: 1,281 mg (99); Chou et al.: 1,228 mg (105)]. Second, we did not use a suprathreshold dose of 1.5 times the morning dose for the on medication condition (106). Finally, our sample was comprised entirely of participants scheduled for STN-DBS surgery and, as a consequence, were medication refractory on-off fluctuators, which is a required criteria for STN-DBS surgery (107).

The main limitation of our study was that many participants were unable to complete all parts of the study. Of the 16 participants with PD, only 7 were completers. There are two likely reasons for this. First, our sample of participants with PD were quite advanced; as such collecting data while off medication and off stimulation proved to be challenging following STN-DBS surgery. Second, the nature of aging is such that it is rare that participants with PD have a single affliction; comorbidities posed a major reason for loss to follow-up. Critically, we were able to use data from 15 of the 16 participants to complete an additional analysis treating missing data as missing at random. The findings of this analysis were similar to the completer analysis indicating that our findings are robust. Another limitation is that all our completers were male, and this could have affected our results. But we think that this was unlikely. This is because two studies that have evaluated the different effects of STN-DBS on females and males found that at 1-year follow-up there were no major differences between males and females on motor function, cognitive and depressive symptoms, and functional status (108, 109). Nevertheless, future studies should consider sex as a biological variable and its implications for STN-DBS treatment.

## Conclusion

The current study sheds light on the differential effect of peripheral-vision and proprioception encoding on reaching performance in participants in PD. Peripheral-vision encoded reaches were faster and more accurate. Moreover, encoding type differentially affected reaching deficits in PD compared to healthy controls: peripheral-vision reaches manifested larger deficits in amplitude. It also sheds light on the effect of the most common treatments, medication and STN-DBS, in the same group of participants with PD. The effect of medication depended on encoding type: medication had no effect on amplitude, but reduced error for both encoding types, and increased velocity only during peripheral-vision encoding. Similarly, the effect of STN-DBS depended on encoding type: STN-DBS increased amplitude for both encoding types, increased error during proprioception encoding, and increased velocity for both encoding types. Finally, we found that STN-DBS was superior to medication with respect to increasing amplitude and velocity, whereas medication was superior to STN-DBS with respect to reducing error. Future studies should examine the volume of tissue activated by STN-DBS and how this affects reaching performance. In addition, the connectivity between the volume of tissue activated and the key cortical nodes in networks underlying memory-guided movements such as occipito-parietal-prefrontal network and the sensorimotor-premotor-cerebellar networks should be assessed to see how this connectivity predicts memory-guided reaching outcomes during vision and proprioception encoding.

## Data availability statement

The statistical dataset supporting the conclusions of this article will be made available by the corresponding author, FD, upon request.

## Ethics statement

The studies involving human participants were reviewed and approved by Northwestern University and Rush University Medical Center Institutional Review Boards and with informed consent from all participants. The patients/participants provided their written informed consent to participate in this study.

## Author contributions

FD and DC conceptualized and designed the study. FD prepared the first draft of the manuscript and conducted the statistical analyses. GP and LV-M recruited the participants, performed preoperative evaluation, intraoperative recordings and assessments, and postoperative stimulation optimization. SS and JR performed the STN-DBS surgery on the participants. FD, YR, QD, and MM conducted testing. FD, YR, TE, RA, QD, and MM processed and analyzed the data. All authors contributed to the interpretation of the data, reviewed, edited, and approved the final manuscript.

## Funding

National Institutes of Health (R01 NS092950, T32 NS047987, and F31 NS120695). The sponsors were not involved in the preparation, review, or approval of the manuscript.

## Conflict of interest

Authors FD and MM received grant support from NIH. Author JR consults for Boston Scientific. Author SS received grant support from NIH, Medtronic, Abbott, and Boston Scientific. Author GP received grant support from NIH and the Parkinson's Disease Foundation. Author LV-M receives honoraria for consulting services/advisory boards from AbbVie, Abbott, Avion, and research support from AbbVie, Abbott, Biogen, Boston Sci., Chase, Medtronic, Neuroderm, Addex, UCB, and NIH. Author DC received grant support from NIH and Michael J. Fox and receives lecture and reviewer fees from NIH. The remaining authors declare that the research was conducted in the absence of any commercial or financial relationships that could be construed as a potential conflict of interest.

## Publisher's note

All claims expressed in this article are solely those of the authors and do not necessarily represent those of their affiliated organizations, or those of the publisher, the editors and the reviewers. Any product that may be evaluated in this article, or claim that may be made by its manufacturer, is not guaranteed or endorsed by the publisher.

## Author disclaimer

The views expressed in this article are those of the authors and do not necessarily reflect the position or policy of NIH.

## Footnotes

^1^MATLAB 2021b, The MathWorks, Inc., Natick, Massachusetts, United States.
