# Supplementary material for: Encoding type, medication, and deep brain stimulation differentially affect memory-guided sequential reaching movements in Parkinson's disease
Source: Front Neurol. 2022 Oct 17;13:980935. doi: 10.3389/fneur.2022.980935 (PMC9618698; doi:10.3389/fneur.2022.980935)
Supplement: Supplementary file 1 [file Data_Sheet_1.docx]

**Supplemental Table 1**. Demographic table (Mean ± Standard Deviation) of completers and non-completers pre STN-DBS surgery

|  | Completers | Non-completers | Difference (p-value) |
| --- | --- | --- | --- |
| Sex (M/F) | 7/0 | 6/3 | NA |
| Age (years) | 65.86 ± 3.89 | 64.11 ± 3.8 | 1.7 (p = 0.384) |
| Disease Duration (years) | 10.71 ± 5.47 | 8.56 ± 4.33 | 2.2 (p = 0.392) |
| MOCA | 27.86 ± 1.95 | 26.78 ± 1.72 | 1.1 (p = 0.259) |
| OFF MDS-UPDRS III | 48.71 ± 10.40^1^ | 52.11 ± 17.03^1^ | -3.4 (p = 0.651) |
| LEDD (mg) | 1319.29 ± 817.95 | 903.89 ± 625.4 | 415.4 (p = 0.268) |

HC, Healthy controls; STN-DBS, Subthalamic nucleus deep brain stimulation; M/F, Male/Female (count); MOCA, Montreal Cognitive Assessment; OFF/ON UPDRS, Movement Disorder Section Unified Parkinson’s Disease Rating Scale Motor Score while OFF/ON treatment; LEDD, Levodopa Equivalent Daily Dose in milligrams

^1^Overnight withdrawal from medication and 3-hour washout for off stimulation

^2^On medication

**Supplemental Table 2**. Comparing the findings from the primary analysis (completers) and the secondary analysis (all available data treating missing data as missing at random) for each research question

| Encoding: peripheral-vision versus proprioception (pre surgery, off-medication) | | | | |
| --- | --- | --- | --- | --- |
|  |  |  | Completers (n=7) | All available data (n=15)^1^ |
|  | **Amplitude (m)** | estimated difference | 0.033 | 0.027 |
|  |  | p-value | <.001 | <.001 |
|  | **Error (m)** | estimated difference | 0.003 | 0.009 |
|  |  | p-value | 0.155 | <.001 |
|  | **Velocity (m/s)** | estimated difference | 0.029 | 0.022 |
|  |  | p-value | <.001 | <.001 |
| Medication: on- versus off- medication (pre-surgery) | | | | |
| **Amplitude (m)** | **Peripheral vision** | estimated difference | 0.003 | 0.004 |
|  |  | p-value | 0.260 | 0.090 |
|  | **Proprioception** | estimated difference | 0.003 | 0.004 |
|  |  | p-value | 0.260 | 0.009 |
| **Error (m)** | **Peripheral vision** | estimated difference | 0.004 | 0.005 |
|  |  | p-value | 0.002 | <.001 |
|  | **Proprioception** | estimated difference | 0.004 | 0.005 |
|  |  | p-value | 0.002 | <.001 |
| **Velocity (m/s)** | **Peripheral vision** | estimated difference | 0.056 | 0.027 |
|  |  | p-value | <.001 | <.001 |
|  | **Proprioception** | estimated difference | 0.002 | 0.003 |
|  |  | p-value | 1 | 1 |
| STN-DBS: on- versus off- STN-DBS (post-surgery, while off- medication) | | | | |
| **Amplitude (m)** | **Peripheral vision** | estimated difference | 0.010 | 0.007 |
|  |  | p-value | 0.003 | 0.010 |
|  | **Proprioception** | estimated difference | 0.001 | 0.007 |
|  |  | p-value | 0.003 | 0.010 |
| **Error (m)** | **Peripheral vision** | estimated difference | 0.004 | 0.005 |
|  |  | p-value | 0.357 | 0.126 |
|  | **Proprioception** | estimated difference | 0.008 | 0.012 |
|  |  | p-value | 0.006 | <.001 |
| **Velocity (m/s)** | **Peripheral vision** | estimated difference | 0.103 | 0.106 |
|  |  | p-value | <.001 | <.001 |
|  | **Proprioception** | estimated difference | 0.103 | 0.106 |
|  |  | p-value | <.001 | <.001 |
| STN-DBS (post-surgery) versus medication (pre-surgery) | | | | |
| **Amplitude (m)** | **Peripheral vision** | estimated difference | 0.007 | 0.001 |
|  |  | p-value | 0.015 | 1 |
|  | **Proprioception** | estimated difference | 0.007 | 0.011 |
|  |  | p-value | 0.015 | <.001 |
| **Error (m)** | **Peripheral vision** | estimated difference | 0.012 | 0.012 |
|  |  | p-value | <.001 | <.001 |
|  | **Proprioception** | estimated difference | 0.012 | 0.012 |
|  |  | p-value | <.001 | <.001 |
| **Velocity (m/s)** | **Peripheral vision** | estimated difference | 0.006 | 0.006 |
|  |  | p-value | 1 | 1 |
|  | **Proprioception** | estimated difference | 0.057 | 0.023 |
|  |  | p-value | <.001 | 0.010 |

Cell with a yellow highlight indicate that the findings from the analysis that used all available data was different from the completer analysis. These differences do not alter our conclusions.

^1^One of the 16 participants in the PD group had demographic and clinical data but did not have any reaching data; therefore, this participant was not included in the analysis that included all available data.
